# The Siah2-HIF-FoxA2 axis in prostate cancer – new markers and therapeutic opportunities

**DOI:** 10.18632/oncotarget.171

**Published:** 2010-09-24

**Authors:** Jianfei Qi, Maurizio Pellecchia, Ze'ev A. Ronai

**Affiliations:** Signal Transduction Program, Sanford-Burnham Medical Research Institute, La Jolla, CA, 92037, USA

## Abstract

Recent studies indicate the importance of the ubiquitin ligase Siah2 in control of more aggressive prostate tumors – namely, neuroendocrine (NE) prostate tumors and prostate adenocarcinoma (PCa) harboring neuroendocrine lesions. Siah2-dependent expression and activity of HIF-1α regulate its availability to form a transcriptional complex with FoxA2, resulting in expression of specific target genes, including Hes6, Sox9 and Jmjd1a, whose co-expression is sufficient for formation of NE tumors and NE lesions in PCa. These studies provide novel markers to diagnose and monitor formation of NE lesions and NE tumors. Furthermore, defining the regulatory axis consisting of Siah2 and HIF-1α/FoxA2 cooperation suggests novel therapeutic modalities to treat these most aggressive forms of prostate cancer. Here we review current understanding of Siah role in control of hypoxia and prostate tumor development and highlight potential approaches for targeting components along Siah-regulated pathways.

## THE E3 UBIQUITIN LIGASE SIAH AND ITS ROLE IN HYPOXIA

Siah is a RING finger E3 ubiquitin ligase that mediates ubiquitination and degradation of substrates important in stress-activated signaling pathways [1, 2]. In humans, Siah has two isoforms, Siah1 and Siah2, which usually target similar substrates. Siah consists of an N-terminal RING finger domain, a central cysteine-rich domain and a C-terminal substrate-binding domain [3]. The important role played by Siah in progression of multiple types of cancer, including lung, pancreatic, breast, prostate and melanoma, is evidenced by inhibition of these tumors in experimental models following attenuation of Siah activity [4-8].

HIF, the master regulator of hypoxia responses, controls diverse transcriptional programs under hypoxic conditions. HIF consists of a heterodimer between α-subunit (HIF-1α or HIF-2α) and β-subunit (HIF-1β). HIF-α levels are regulated by PHD (prolyl-hydroxylase)-mediated hydroxylation of two proline residues that leads to VHL-dependent degradation. In addition HIF-α activities are regulated by FIH (factor inhibiting HIF-1α)-mediated asparagine hydroxylation that inhibits HIF transcriptional activity by disrupting p300/CBP binding. Siah can elicit ubiquitination and degradation of PHD1/3 and FIH, thus stabilizing HIF-α and promoting its transcriptional activity [9, 10]. In addition, Siah has been shown to regulate the stability of HIPK2 [11, 12], a transcriptional repressor of the HIF-1α gene [13]. Thus, via three independent pathways, Siah contributes to HIF-α transcription, stability and activity. Consistently, cancer cells lacking Siah or expressing a Siah inhibitory peptide exhibit reduced levels and activity of HIF-α [7, 8].

## SIAH IN PROSTATE CANCER DEVELOPMENT AND PROGRESSION

Prostate cancers are the most common malignancy in American men [14]. Hence, understanding mechanism underlying the development and progression of prostate cancers offers possible means for monitoring and therapy. Our recent study demonstrates the importance of the Siah-HIF axis in development of prostate NE tumors and NE lesions in human prostate adenocarcinomas, which are among the more aggressive prostate tumors. Although prostate NE carcinomas are rare (< 5%), over 30% of human prostate adenocarcinomas show expression of NE markers (e.g. NSE, chromogranin), often referred to as NE differentiation (NED) or the NE phenotype. NED of prostate cancers is associated with tumor progression, resistance to therapy (such as androgen-deprivation therapy, radiotherapy, chemotherapy), and poor prognosis [15-18].

To evaluate a potential role for Siah in prostate tumor progression, we crossed *Siah*-deficient (*Siah2*^−/−^ or *Siah1a*^+/−^*::Siah2*^−/−^) mice with the TRAMP mouse model. Prostate-specific expression of SV40 T antigen in the TRAMP mice leads to formation of two major types of lesions: prostate NE carcinoma and atypical hyperplasia (AH). Siah-deficiency significantly reduced formation of prostate NE carcinoma in TRAMP mice, while AH lesions were less affected. *Siah*-deficient TRAMP NE tumors showed reduced HIF-1α levels, decreased cell proliferation, increased cell death, but no apparent effect on vascular density. To confirm that impaired NE tumor formation is due to Siah's effect on HIF, we transfected TRAMP cells with a Siah inhibitory peptide and observed reduced HIF-1α levels and inhibited tumor formation in nude mice. Conversely, forced re-expression of HIF-1α in TRAMP cells expressing Siah inhibitory peptide partially restored these cells' ability to form xenograft tumors, indicating that Siah regulation of HIF is essential for development of NE prostate tumors in the TRAMP model. Consistent with our study, *AhR* (aryl hydrocarbon receptor)-null TRAMP mice showed increased formation of NE carcinoma but not of AH [19]. Since HIF-1β dimerizes with both AhR and HIF-1α, lack of AhR may promote the formation of HIF-1α/HIF-1β dimers, thereby enhancing HIF activity and formation of NE carcinoma [20]. These observations underscore the importance of HIF in the development of prostate NE carcinoma in the TRAMP model.

The finding that NE tumors can no longer develop in the absence of Siah, combined with observation of reduced HIF-1α expression, led us to explore the possibility that HIF-1α activity is required for the formation of NE phenotypes. In addition to HIF-1β, HIF-α can also interact with other transcription factors such as β-catenin, Notch, c-Myc [21-24]. Thus, we tested the possibility that HIF may elicit a tissue-specific transcriptional program by interacting with factors expressed in NE tumors. Among transcription factors that are important for the formation of NE tumors in the TRAMP model is FoxA2 [25], a member of the Fork-head family of transcription factors consisting of FoxA1, FoxA2 and FoxA3 [26]. Although FoxA1 is expressed in both NE tumors and AH, FoxA2 is only expressed in NE tumors in the TRAMP or LADY model [25, 27]. Using an HRE-luciferase assay that serves as a marker for HIF activity, we discovered FoxA2/HIF transcriptional synergy [7]. Notably, FoxA2 interacted with HIF-1α via the N-terminal domains of each protein, promoting recruitment of p300 to specific HIF target genes and activating transcription. Consistent with the possibility that p300 can be recruited by specific HIF transcriptional program to induce a select set of proteins is the reports that MEFs isolated from mice harboring a mutant form of p300/CPB, which cannot interact with HIF-α, or MEFs derived from FIH KO mice showed impaired expression of only a subset of HIF target genes under hypoxia [28, 29], supporting the notion that p300/CBP is required for expression of specific HIF target genes. Cooperation between FoxA2 and HIF-1α resulted in transcriptional activity that was substantially greater than that seen by HIF alone. Microarray analyses identified approximately 40 genes (such as, Hes6, Sox9, Jmjd1a, and Plod2) that were co-regulated by HIF and FoxA2 in TRAMP cells, pointing to a specific transcriptional program that is regulated by this cooperation. Notably, some of the proteins that are regulated by HIF-1α-FoxA2 cooperation were reported to be HIF targets. In addition, FOXA binding sites have been found in some of the genes co-regulated by HIF and FoxA2 [7], providing additional explanation for HIF/FoxA2 synergy on specific HIF targets.

Following the identification of HIF/FoxA2 target genes, we assessed their importance in development of prostate NE tumors. In these studies we have focused on assessing three genes: Hes6, Sox9 and Jmjd1a, which were implicated in prostate tumors, and were shown (by us and others) to be regulated by HIF. To this end, TRAMP cells were engineered to express Siah-inhibitory peptide or FoxA2 shRNA. These modified cells showed reduced tumor-forming capacity in an orthotopic prostate tumor model. Significantly, co-expression of Hes6, Sox9 and Jmjd1a partially rescued tumorigenic capacity in the presence of Siah-inhibitory peptide, confirming the significance of the pathway in prostate NE tumor development. Notably, re-expression of Hes6, Sox 9 or Jmjd1a individually was not sufficient to rescue tumorigenesis in TRAMP cells in which either HIF or FOXA2 was inhibited, indicating that multiple genes regulated by HIF/FoxA2 are required for development of prostate NE tumors. We expect that co-expression of additional genes identified to be regulated by the HIF/FoxA2 program will increase the rescue of these tumors.

## SIAH CONTROLS THE FORMATION OF NE LESIONS IN ADENOCARCINOMA OF THE PROSTATE

The specific transcriptional program mediated by HIF and FoxA2 also plays a key role in development of NE lesions (aka NED or NE phenotype) in human prostate adenocarcinoma. As a model to study NE phenotype in vitro we used the human prostate adenocarcinoma cell line CWR22Rv1. Notably, when maintained under hypoxia for few days these cells show upregulation of the NE marker NSE and protrusion of neurite-like structures [7]. Correspondingly, orthotopic prostate tumors formed in mice by these human PCa cells exhibit high NSE levels in necrotic regions, which are known to be highly hypoxic. Thus, hypoxia appears to be important for prostate cancer NE phenotypes in vitro and in vivo. The in vitro NE phenotype of the CWR22Rv1 cells was Siah/HIF and FoxA2 dependent and could be rescued upon co-expression of the Hes6, Sox9 and Jmjd1a genes. Orthotopic injection of CWR22Rv1 cells expressing Siah-inhibitory peptide or FoxA2 shRNA did not alter their ability to develop tumors but rather abolished their propensity to metastasize and inhibited the appearance of NE phenotypes. Co-expression of Hes6, Sox9 and Jmjd1a was sufficient to rescue metastasis, indicating the importance of the NE phenotype the metastatic capacity of CWR22Rv1 cells. Consistent with these observations, immunohistochemistry staining of a prostate cancer TMA and analysis of a microarray data revealed higher levels of Siah2, FoxA2, Hes6, Sox9, Jmjd1a and NSE in high-grade human PCa (Gleason score 4 and 5) and in metastatic prostate cancers [7]. These findings underscore the importance of the Siah2/HIF/FoxA2 axis in PCa NE phenotype, tumor progression and metastasis.

Among other PCa cells that exhibit NED in culture are LNCaP cells. Although diverse stimuli, such as IL-6, radiation, or androgen deprivation, can induce NED in cultured LNCaP cells [30-32], androgen deprivation is the main inducer of NED in the mouse model [33-36]. Importantly, the incidence of NED is elevated in prostate cancer patients after androgen deprivation therapy [37]. Androgen deprivation causes damage of blood vessels in the prostate or prostate tumors with a consequent increase in hypoxia [38-40], which may contribute to the NED of prostate cancers. This possibility is consistent with our observation that NED occurs primarily in more hypoxic regions.

Recent evidence indicates the presence of a small population of self-renewing and multipotent cells within solid tumors, termed cancer stem cells (CSCs) or tumor-initiating cells [41]. These cells share many features with somatic and embryonic stem (ES) cells and are believed to drive tumor progression, metastasis and resistance to therapy. NED lesions of prostate cancers have been shown to express the stem cell marker Oct4A and the CSC marker CD44 [42, 43], implying that the NED lesions may harbor prostate cancer stem cells. In the TRAMP model, prostate NE tumors are believed to be derived from prostate stem cells in the mouse proximal prostate gland [25, 44]. The importance of Siah/HIF/FoxA2 axis in the formation of TRAMP NE tumor and NED of human prostate cancers suggests this signaling pathway may play a key role in the prostate stem cell function.

## POSSIBLE CLINICAL RELEVANCE

### A. Diagnostics

Based on our identification of novel proteins associated with the NE lesions and NE prostate tumors we assessed expression of Hes6 and Sox9 in tissue specimens. These studies, performed by IHC, demonstrated the ability to detect NE lesions in the high-grade prostate cancers. Given the difficulty of detecting NE lesions, the availability of new markers should promote development of additional antibodies and improved methods to detect these lesions, which are associated with poor prognosis. The ability to better define NE tumors and lesions at early disease stages should also impact treatment options and provide tools necessary to assess treatment efficacy. The availability of novel NED markers should also promote research into different types of NED (i.e., staining of the NE markers in single cells, cell clusters, or diffuse areas within PCa) and facilitate characterization of possible prostate CSCs within these lesions.

### B. Therapeutic Strategies

**Figure 1:**
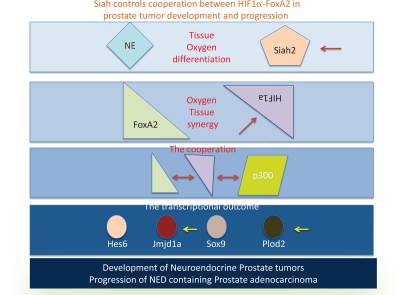
Siah2 controls the HIF-1α levels in the hypoxic condition, allowing the availability of HIF-1α for interaction with the NE-specific transcription factor FoxA2, which is expressed in prostate EN tumor or the NE lesions of PCa FoxA2/HIF-1α interaction together with the recruitment of p300/CPB promotes the transcription of select HIF targets such as Hes6, Sox9, Jmjd1a and Plod2. The selective transcriptional program elicited by HIF/FoxA2 is required for the development of prostate NE tumors and formation of NED lesions in the PCa. Arrows point to components in this pathway that can be used for diagnosis and therapeutic targeting.

As shown in Fig 1, the findings that Siah controls levels and activity of HIF-1α, which cooperates transcriptionally with FoxA2 to promote NE tumor development or formation of NED of human prostate cancers, provide a rationale for targeting the Siah/HIF/FoxA2 axis as a new therapeutic modality. Such an approach is supported by the following: (1) the presence of NED in PCa is associated with resistance to current therapies, including androgen deprivation therapy, radiotherapy and chemotherapy; and (2) androgen deprivation therapy or radiotherapy may induce NED of prostate cancers. Therefore, targeting the Siah/HIF/FoxA2 axis should inhibit the NE phenotype, sensitizing tumors to traditional prostate cancer therapy.

**Figure 2:**
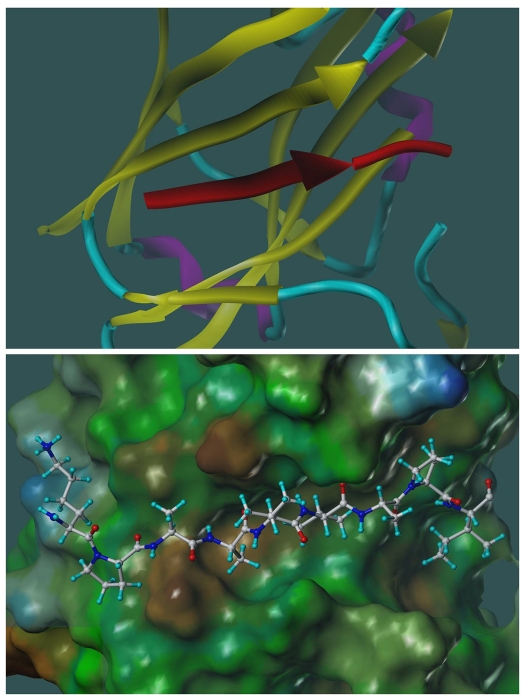
Molecular models representing the X-ray structure of SIAH1 in complex with a SIP derived peptide of sequence EKPAAVVAPITTG (PDB-ID 2A25;) A similar docked geometry was observed in another –X-ray study with SIAH1 in complex with a 24 amino acids Phyllopod peptide (PDB-ID 2AN6). Top panel: ribbon representation of SIAH1 yellow, β-strands; cyan, loop regions, magenta, α-helical regions; the SIP peptide completes a β-strand and it is depicted in red. Bottom panel; SIAH-SIP complex is displayed in the same orientation as in the top panel, but SIAH1 is shown as a surface representation, color coded according to a molecular lipophilicity potential (brown more lipophilic; green, neutral; blue, less lipophilic). The peptide is shown as a ball-and-stick representation. The figure was prepared with MOLCAD as implemented in Sybyl (Tripos, St.Louis).

Three components in the newly discovered pathway could be targeted as potential therapies. Of the three, Siah is likely the best candidate, since its loss abolishes formation of TRAMP NE tumors and restoring HIF expression in such tumor cells only partially (30%) rescues formation of NE tumors. Notably, FoxA2 inhibition is less effective in attenuating NE tumor formation. As a RING finger E3 ubiquitin ligase Siah could be targeted in several ways. The least favorable is targeting its RING domain, given its similarity with the RING domain present in other E3 ligases. A Siah domain that would allow more specific inhibition is the substrate-binding groove, which is required for binding with adaptors or substrates containing a peptide motif RPVAxVxPxxR, that mediates the interaction of Siah protein with a range of protein partners [45, 46]. A high-affinity interaction with a peptide from the cytoskeletal protein plectin-1 (residues 95–117) was identified with an apparent Kd of about 29 nM [45]. Given availability of structural data concerning the Siah2-adaptor binding complex, and given the available crystal structure of Siah2 one can envision use of structure-based design of peptides and peptide-mimetics that selectively interfere with Siah2 association with selective substrates or adaptor proteins (Figure 2). Structure-based design combined with high throughput screening may allow identification of small molecules to block the Siah substrate-binding domain (SBD). Supporting this approach is the observation that a 22 aa Siah inhibitory peptide can bind to the Siah SBD and reduce HIF levels under hypoxia [47]. A structure-based approach may be complemented by assessing the effect of inhibitors on Siah2 self-ubiquitination, a property inherent to RING finger E3 ligases. As a proof of principle, we recently established a Meso-Scale-based assay and, out of 2000 compounds, identified Menadione as a Siah2 inhibitor. Menadione treatment inhibited HIF levels in cultured cells, increased expression of direct Siah2 targets, and inhibited formation of melanoma xenografts [48]. Given that inhibition of Siah2 provides an unprecedented opportunity for the discovery of novel anti-cancer agents we believe that further drug discovery efforts are justified.

Several inhibitors directed against HIF have been recently developed [49-51]. It is of importance to assess their effects in prostate tumor models, including those used in our recent studies. A more specific target emerging from our studies, however, is the HIF/FoxA2 structural interface. The crystal structure of this complex or domain should provide information required to develop highly specific inhibitors. Although these inhibitors may not be as potent as HIF or Siah inhibitors, they could be more specific to the NE phenotype. Notably, targeting protein-protein interaction has been a less favorable approach for drug design; thus development of more advanced technologies is required to make this task more feasible. Structure-based approaches will likely dominate the search for Siah and HIF/FoxA2 inhibitors.

Inhibiting specific HIF-FoxA2 targets is also a possibility. Some of these proteins identified in our studies, such as Jmjd1a and Plod2, exhibit intrinsic enzymatic activity. Studies are underway to assess the consequence of their selective inhibition on NE tumors and NED lesions in culture and in vivo. Of consideration is the notion that unless inhibitors are targeted specifically to NE cells, blocking these enzymes might promote pleiotropic effects.

Targeting components along the newly discovered Siah/HIF/FoxA2 axis, which functions in formation of prostate cancer NE phenotypes and is potentially associated with the prostate cancer stem cells, should complement current diagnosis and therapies for prostate cancer patients whose tumors harbor NE markers.
